# Supplementary material for: Utilizing Genome-Wide mRNA Profiling to Identify the Cytotoxic Chemotherapeutic Mechanism of Triazoloacridone C-1305 as Direct Microtubule Stabilization
Source: Cancers (Basel). 2020 Apr 2;12(4):864. doi: 10.3390/cancers12040864 (PMC7226417; doi:10.3390/cancers12040864)
Supplement: Supplementary file 1 [file cancers-12-00864-s001.zip › cancers-743562-SUPPL-FIANL/cancers-743562-supplementary figures.pdf]

# Supplementary Materials: Utilizing Genome-Wide mRNA Profiling to Identify the Cytotoxic Chemotherapeutic Mechanism of Triazoloacridone C-1305 as Direct Microtubule Stabilization

**Jarosław Króliczewski, Sylwia Bartoszewska, Magdalena Dudkowska, Dorota Janiszewska, Agnieszka Biernatowska, David K. Crossman, Karol Krzymiński, Małgorzata Wysocka, Anna Romanowska, Maciej Baginski, Michal Markuszewski, Renata J. Ochocka [1], James F. Collawn, Aleksander F. Sikorski, Ewa Sikora and Rafal Bartoszewski**

## Synthesis of C-1305

*2,6-Dichloro-3-Nitrobenzoic Acid (1)*

100 g (0.523 mol) of 2,6-dichlorobenzoic acid was dissolved in 2000 mL of concentrated sulfuric(VI) acid (p.a., 96% w/w, *Stanlab*, Poland) and the solution was placed in an ice bath. 40 mL of nitric(V) acid solution (p.a., 65% w/w $HNO_3$, *Eurochem BGD*, Poland) was introduced in small portions to the mixture set at magnetic stirrer, controlling the temperature, which should not exceed 40 °C during the reaction. After adding of $HNO_3$ (ca. 30 min.), the mixture was stirred for 3 h at 45–50 °C, and then the mixture was poured into a large excess of ice-water mixture. The white precipitate was filtered using Buchner glass funnel, washed with distilled water and dried. 2,6-Dichloro-3-nitrobenzoic acid was obtained in a near theoretical yield (m.p. = 142–144°C (uncorrected); TLC: $R_F$ = 0.19, $SiO_2$, $CHCl_3$/methanol = 5/1 v/v).

*6-Chloro-3-Nitro-2-{[4-(Benzyloxy)Phenyl]Amino}benzoic Acid (2)*

A solution of 30.6 g (130 mmol) g 4-(benzyloxy)aniline hydrochloride (98%, Sigma-Aldrich, USA) in 35 mL of triethylamine was added to 23 g (97 mmol) of 2,6-dichloro-3-nitrobenzoic acid (**1**) dissolved in 50 mL of ethanol and heated under reflux (80 °C) for 48 hours. The contents were then evaporated at reduced pressure and the residual powder was dissolved in 500 mL of chloroform and stirred with 500 mL of 1 M NaOH. The aqueous layer was separated and orange precipitate of 6-chloro-3-nitro-2-{[4-(benzyloxy)-phenyl]amino}benzoic acid was obtained by addition of 2 M HCl solution (final pH = 2). The resulting pale orange product was filtered off, washed with cold water and dried at room temperature (yield = 55–60%, m.p. = 175.2 ± 0.01°C (*M-565 Melting Point, Buchi*, Switzerland), $R_F$ = 0.54, $SiO_2$, $CHCl_3$/methanol = 5/1 v/v).

*7-Benzyloxy-1-Chloro-4-Nitroacridin-9-(10H)-One (3)*

40 g (92 mmol) of 6-chloro-3-nitro-2-{[4-(benzyloxy)phenyl]amino}benzoic acid (**2**), 80 mL of phosphorus oxychloride (p.a., *Sigma-Aldrich*) in 200 ml of chloroform was heated under reflux (100 °C) for 1.5 hours. Progress of the reaction was monitored by thin-layer chromatography (Silica gel 60 (Merck) in chloroform/methanol (10/1 v/v) solvent system. The reaction mixture was then cooled down and left in the fridge overnight. A red precipitate of 7-benzyloxy-1-chloro-4-nitroacridin-9-(10*H*)-one was filtered off and dried (yield 73%, m.p. = 216.9°C, $R_F$ = 0.93 ($SiO_2$, $CHCl_3$)).

*1-Chloro-7-Hydroxy-4-Nitroacridin-9(10H)-One (4)*

35 g (97 mmol) of 7-benzyloxy-1-chloro-4-nitroacridin-9(10*H*)-one (**3**) was suspended in 700 mL of glacial acetic acid (p.a., *Sigma-Aldrich*, USA), then 30 mL of methanesulfonic acid (p.a., *Sigma-Aldrich*, USA) was added and the contents were heated under reflux (100°C) for 7 hours. The reaction mixture was then cooled down and the red precipitate of 1-chloro-7-hydroxy-4-nitroacridin-9(10*H*)-one was filtered off, washed with acetic acid, cold ethyl ether and dried (yield: 75%, m.p. = 334.9°C).

*4-Amino-1-Chloro-7-Hydroxyacridin-9(10H)-One (5)*

2.81 g (9,72 mmol) of 1-chloro-7-hydroxy-4-nitro-(10*H*)acridin-9-one was suspended in 20 mL methanol and 15.6 g (69.1 mmol) of tin(II) chloride ($SnCl_2 \times 2H_2O$) in 12 ml concentrated HCl and was added. The mixture was heated to reflux (80 °C) for 5 h. The reaction mixture was then cooled down and poured into cold water (200 mL). The pale yellow precipitate of the product was filtered off, washed twice with cool water and crystallized from DMF-$H_2O$ to give 1-chloro-4-amino-7-hydroxyacridin-9-(10*H*)one as yellow needles (yield: 60–65%, m.p. = 224.1±0.40°C, $R_F$ = 0.66 ($SiO_2$, ethyl acetate).

*5-Chloro-7-Hydroxy-6H-[1,2,3]Triazolo[4,5,1-de]Acridin-6-One (6)*

A suspension of 4-amino-1-chloro-7-hydroxyacridin-9(10*H*)-one (5.89 g, 22.7 mmol) in 80 mL of concentrated hydrochloric acid (p.a., 36% HCl w/w, Stanlab, Poland) was stirred at room temperature for 30 min. A solution of nitrate(III) sodium (p.a. $NaNO_2$, POCh, Poland) (2.6 g, 37.7 mmol) in 60 ml of water was added to the suspension in small portions and the resulting mixture was stirred at room temperature for 4 hours. 20 mL of water was then added to the reaction mixture, the precipitate was filtered off, washed with water, and crystallized from DMF-$H_2O$ mixture to give 5-chloro-7-hydroxy-6*H*-[1,2,3]-triazolo[4,5,1-*de*]acridin-6-one as yellow-green needles (yield = 85%, m.p. = 289.50±0.50°C, $R_F$ = 0.90 ($SiO_2$, ethyl acetate)).

*7-Hydroxy-5-{[3-(Dimethylamino)Propyl]Amino}-6H-[1,2,3]Triazolo[4,5,1-de]Acridin-6-One (C-1305)*

0.407 ml (3 mmol) 3-(dimethylamino)propan-1-amine (*Sigma-Aldrich*) was added to a suspension of 0.27 g (1 mmol) of 7-hydroxy-5-chloro-6*H*-[1,2,3]-triazolo[4,5,1-de]acridin-6-one (**6**) in 5 mL of anhydrous dimethylformamide (DMF, *Sigma-Aldrich*). The contents were mixed with heating at 60°C for 4 hours. The progress of the reaction was monitored by TLC on Silica gel 60 plates (*Merck*) in chloroform-methanol-25% solution of ammonia (5/1/0.1 v/v/v) solvent system. The reaction mixture was then diluted with chloroform and to remove the excess of amine the one was washed with a diluted solution of HCl and next with water. The organic layer was dried over anhydrous sodium sulphate(VI), the solvent was evaporated under reduced pressure. The residue was purified by gravitational column chromatography ($SiO_2$), using gradient solvent system (chloroform/methanol = 50/1–5/1 v/v, followed by chloroform/methanol/25% solution of ammonia = 5/1/0.1 v/v/v (yield = 85%, m.p. = 220.70 ± 0.78 °C (lit.: 228–230°C [1]); $R_F$ = 0.53 ($SiO_2$, $CHCl_3$/methanol/25% solution of ammonia = 5/1/0.1 v/v/v)).

Synthesis route for **C-1305** is summarized in Fig. S1 and its representative analyses are presented in Figs S2–S5.

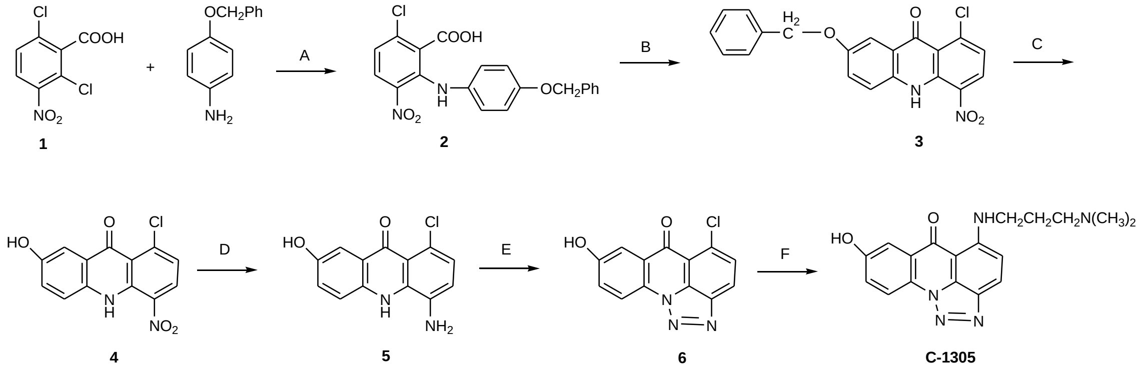


**Figure S1.** Scheme of synthesis of compound C-1305 performed for this work. Step A: $Et_3N$, EtOH, reflux, 48 h; B: excess $POCl_3$, reflux, 1.5 h; C: methanesulfonoic acid, glacial AcOH, reflux, 7 h; D: $SnCl_2 \times 2H_2O$, HCl/MeOH, reflux, 5 h; E: $NaNO_2$, HCl conc., RT, 4 h; F: NH2(CH2)3N(CH3)2, anh. DMF, 60 °C.

| **Sample ID:** | C-1305 | | |
|---|---|---|---|
| Automatic: | Left | Center | Right |
| | 221.2 °C | | 220.1 °C |
| | Average: | 220.7 °C | |
| | Standard deviation: | 0.78 °C | |

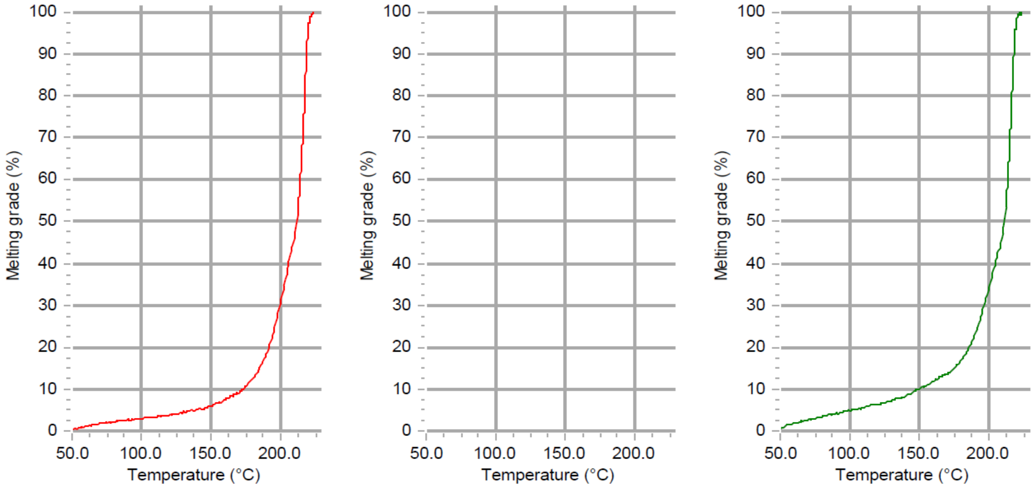


**Figure S2.** Melting profiles of compound **C-1305** (start temperature = 50°C, temp. gradient = 5 °C/min (*M-565 Meling Point, Buchi*, Switzerland).

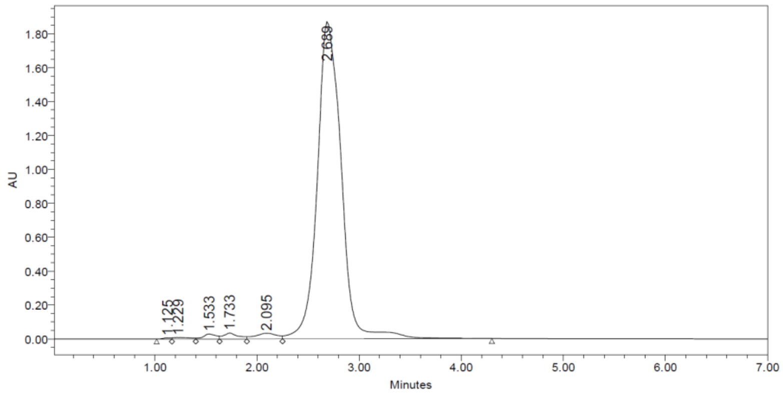


**A**

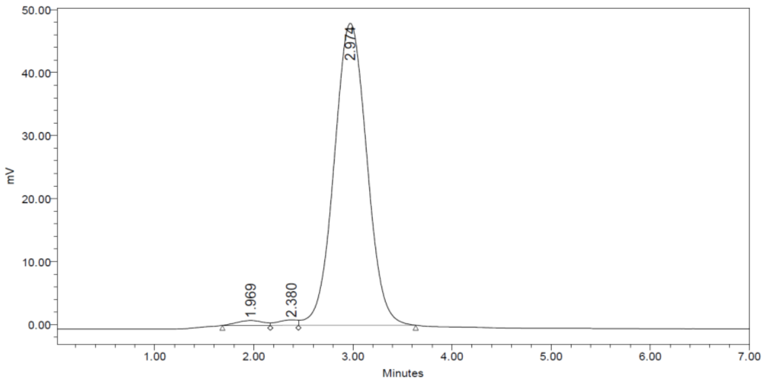


**B**

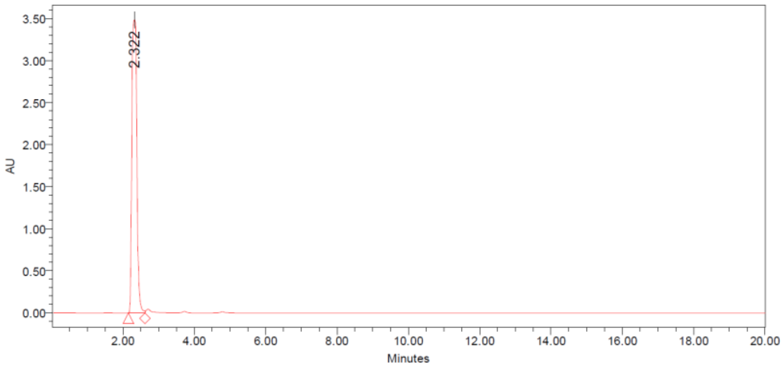


**C**

**Figure S3.** RP-HPLC chromatograms of purified compound C-1305 (neutral form). Experiment setup: Waters HPLC set (W600 pump, W2487 Dual Wavelength Absorbance Detector ($\lambda$ = 265/360 nm), W474 Fluorescence Scanning Detector. Graph **A**: absorbance at 360 nm, Graph **B**: fluorescence at $\lambda_{ex}$/($\lambda_{em}$ = 400/462 nm; mobile phase: 20 mM $KH_2PO_4$ / 20 mM $K_2HPO_4$ / 1.0 methanol = 0.5/0.5/1.0 v/v/v, flow rate = 1 mL/min.; stationary phase: Phenyl C6 column (4.6 × 150 cm, *Phenomenex*, USA). Graph **C**: absorbance at 254 nm, mobile phase: $H_2O$ + 0.1% TFA/methanol = 1/1 v/v, flow rate = 1 mL/min.; stationary phase: Symmetry C18 column (4.6 × 150 cm, Waters, USA).

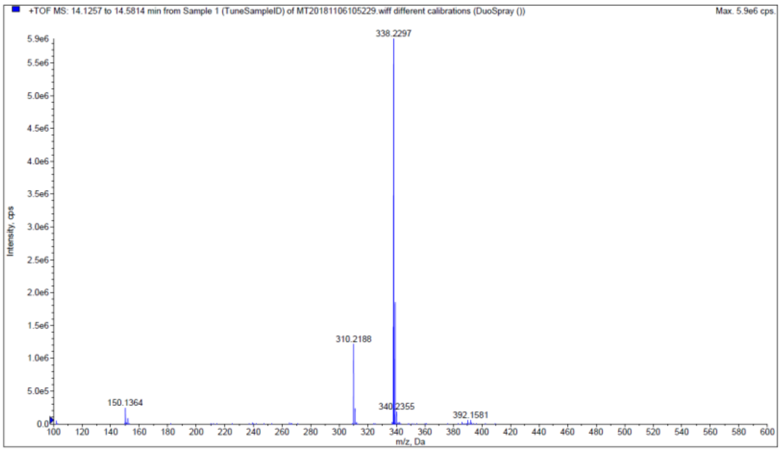


**A**

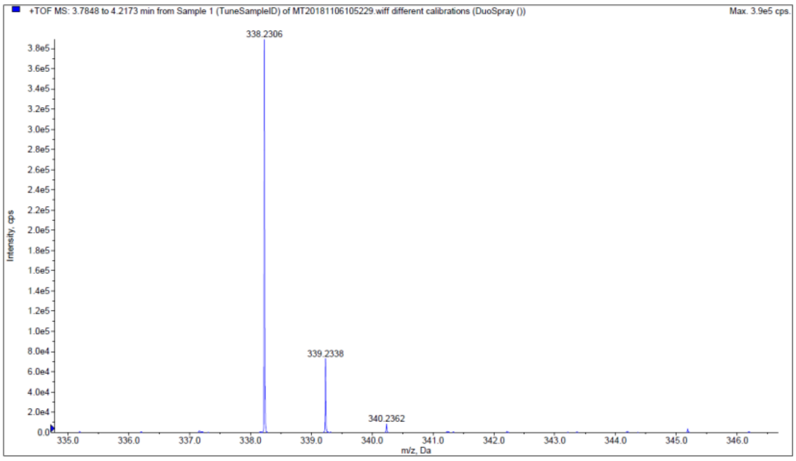


**B**

**Figure S4.** Mass spectra (positive ionization mode) of compound C-1305. Graph **A**: wide range, parent cation m/z = 338.23 $(M+1)^+$; Graph **B**: narrow range with the isotope pattern of the main signal denoted.

Apparatus: QTRAP Quadrupole Time-Of-Flight (QTOF) mass spectrometer (*AB SCIEX*, Canada), electrospray (ESI) ionization.

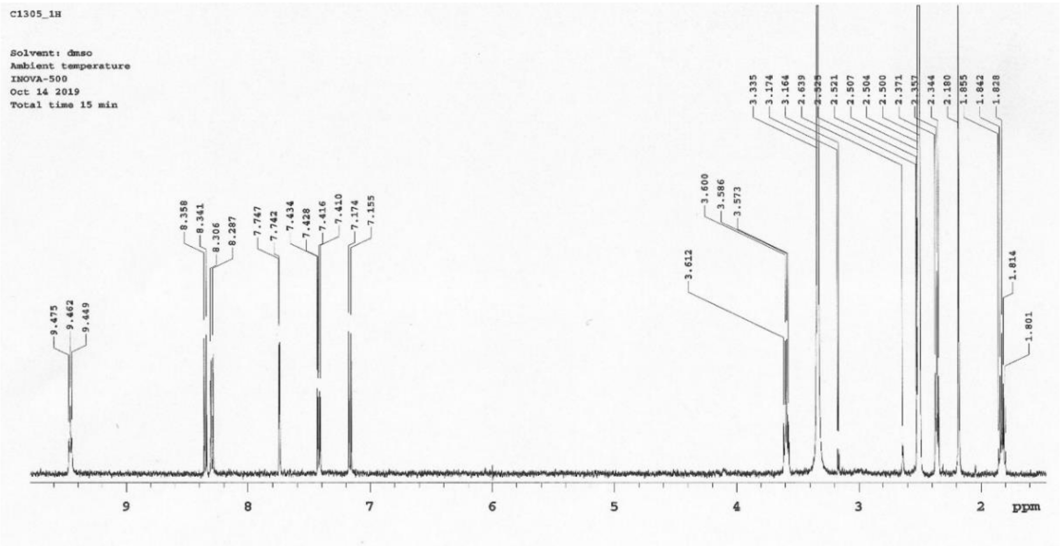


**Figure S5.** $^1$H NMR spectrum (*Varian Unity Inova 500*, 500 MHz) of compound C-1305 ($CDCl_3$, room temperature).

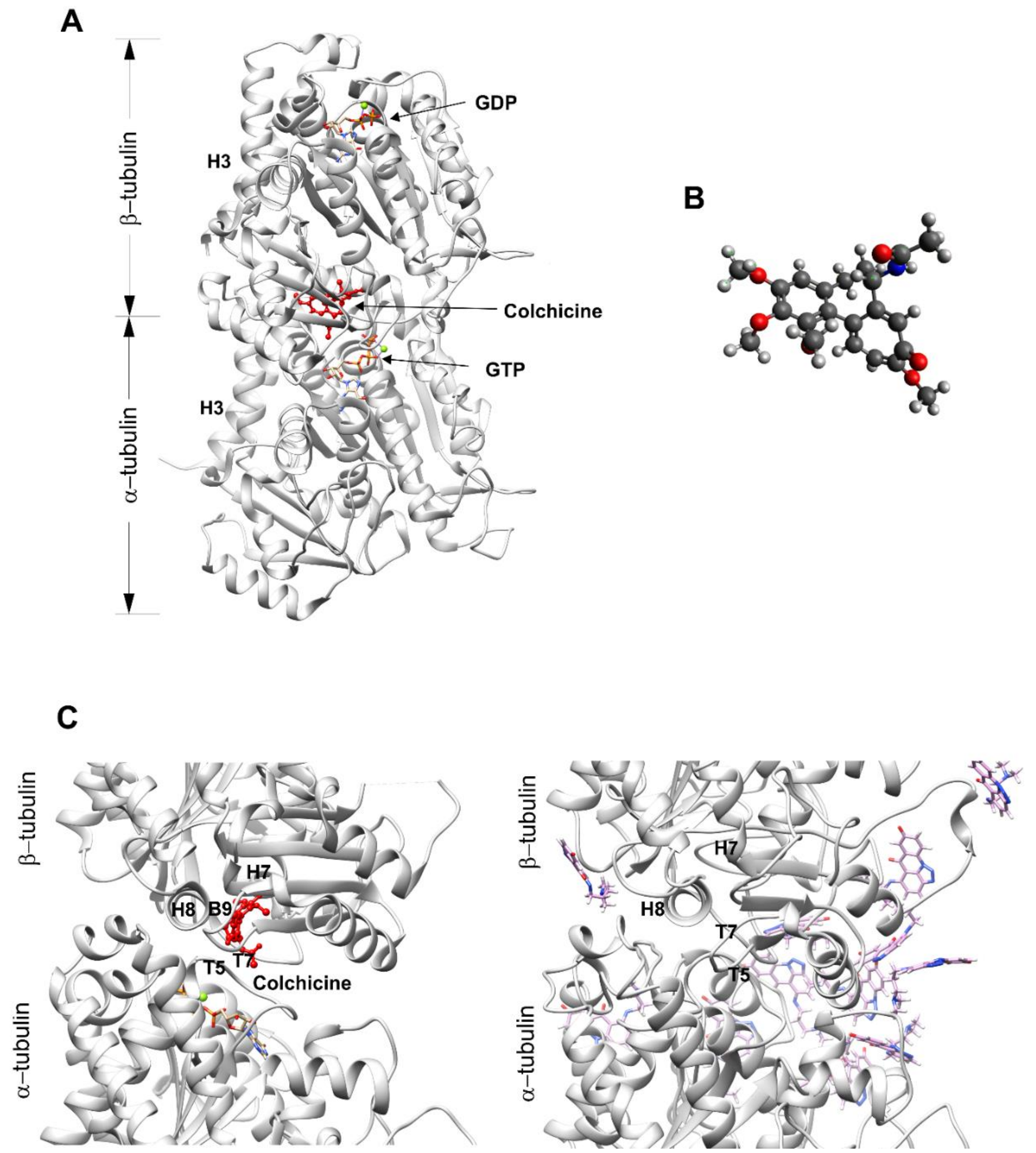


**Figure S6. (A)** The α-tubulin and β-tubulin heterodimer structure. Colchicine molecule is shown in red. The GTP and GDP are shown using a colored ball and stick models. Mg is shown as a green ball. The data for tubulin dimer with complexed colchicine molecule was obtained by engineering the crystal structure of colchicine-bound tubulin, (4O2B, PDB). **(B)** The 3D structure of colchicine generated as described under the Materials and methods section. The red, grey, black and blue colors represent oxygen, hydrogen, carbon and nitrogen atoms, respectively. **(C)** Comparison of a close-up of the representative snapshots of the colchicine binding packet and simulations depicting the interactions of C-1305 at the luminal side of tubulin. Regions of colchicine binding pocket are consist of T5 and T7 loop the cylindrical H7 and H8 helices with and B9 sheet [3].

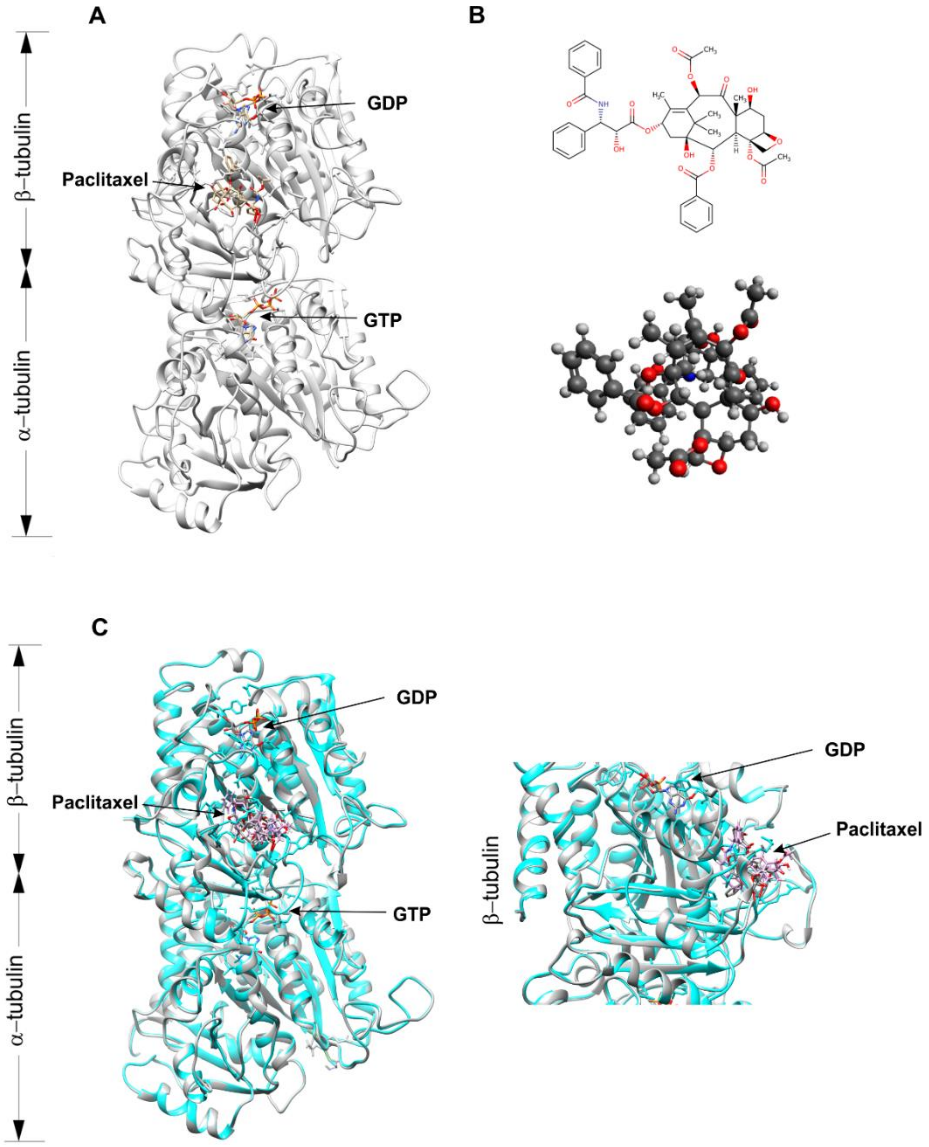


**Figure S7. (A)** The α-tubulin and β-tubulin heterodimer structure**.** The data for tubulin dimer with complexed paclitaxel molecule was obtained by engineering the crystal structure of paclitaxel-bound tubulin, (1TUB, PDB), Paclitaxel, GTP and GDP are shown using a colored stick models (**B**) Chemical formula (top panel) and predicted 3-dimensional (3D) structure of paclitaxel. The mol2 file of paclitaxel molecule with all hydrogens and 3D coordinates was generated using UCSF Chimera, and MarvinSketch. The program Avogadro version 1.2.0 was used to perform geometric optimization of unbound C-1305 with 500 steps of steepest descent using the MMFF94 force field.2 [42] (**C**) Tubulin dimmer crystal structure (1TUB) superimposed with the computed tubulin dimmer allosteric structure. The ligand is shown in stick. Protein residues shown in stick (grey, 1TUB; pink, model). Protein backbone shown in cartoon (light grey, 1TUB; cyan, model). Comparison of a close-up of the representative snapshots of the paclitaxel binding packet (1TUB and simulations model).The graphics was generated using UCSF Chimera [43].

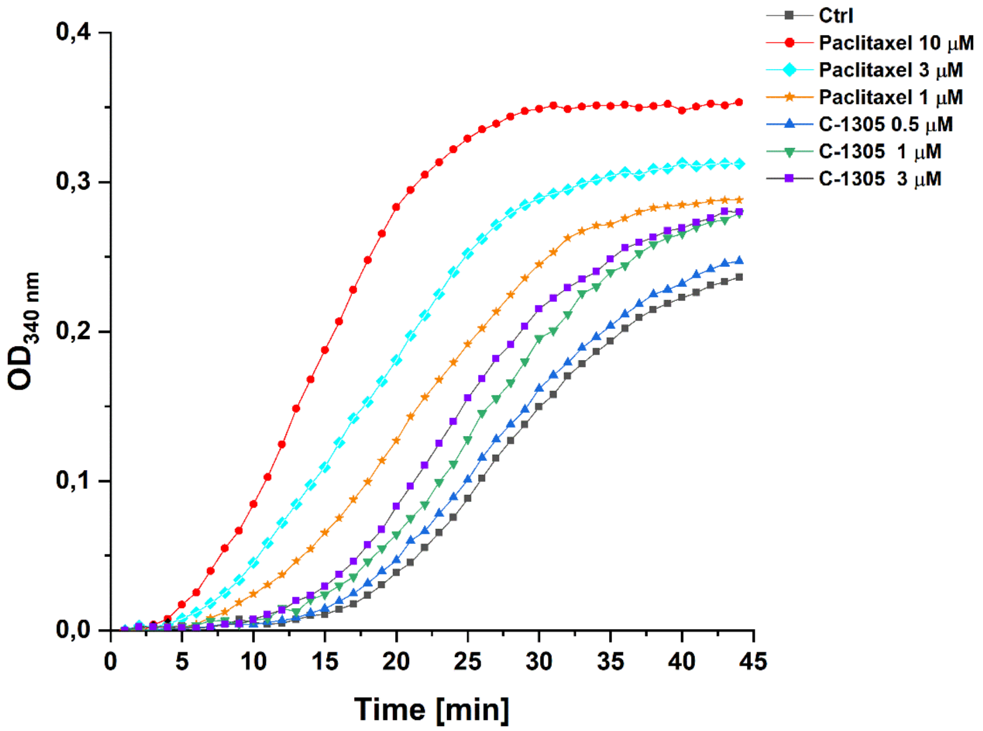


**Figure S8.** Dose-dependent effect of C-1305 or paclitaxel on in vitro microtubule formation. The level of the polymerization was measured by an increase in turbidity (OD) at 340 nm (see Materials and methods section).

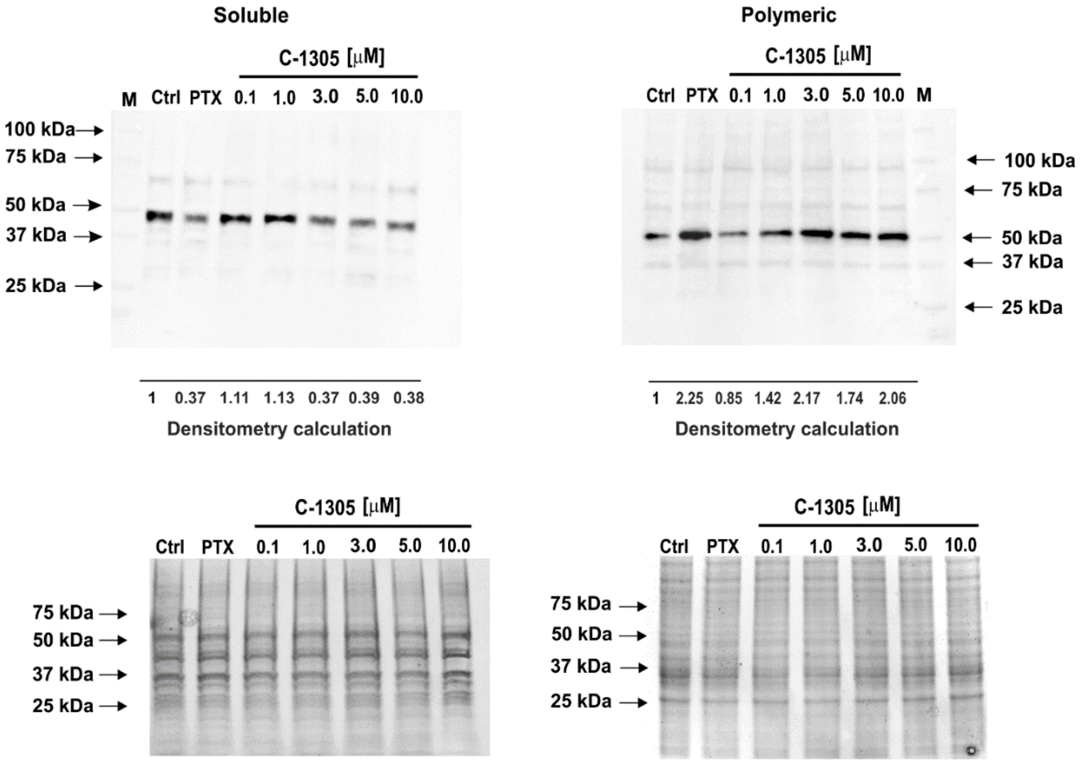


**Figure S9.** C-1305 stabilized the polymeric tubulin and changed the microtubule network in A549 cells. (A) SDS-PAGE (bottom panel) and Western blot analysis (top panel) of soluble and polymeric tubulin fraction in A549 cells after C-1305 treatment. Western blot analysis of soluble (S) and polymeric (P) tubulin fraction using rabbit primary antibody against β-tubulin (ab18207, Abcam) and HRP-conjugated secondary antibodies (Bio-Rad) and detected using ECL (Amresco). The molecular weight markers are indicated by the arrows on the left (Precision Plus Protein™ Kaleidoscope™

Prestained Protein Standards BioRad, #1610375). Densitometry was performed using Image Lab software v. 4.1 (Bio-Rad) and Gel-Pro Analyzer v. 4.0 (Media Cybernetics L.P), and the amount in each band was calculated in relation to the corresponding band of Ctrl lane. The bottom panel shows the total protein loading controls SDS-PAGE of soluble (S) and polymeric (P) tubulin fraction isolated from A549 cells after 6 h of treatment.

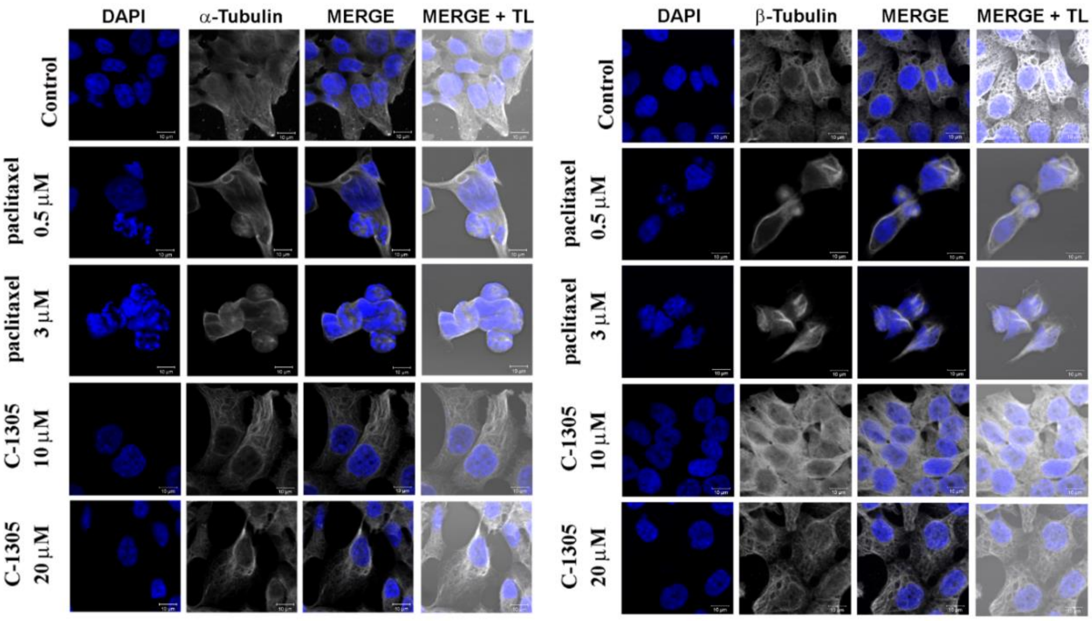


**Figure S10.** The C-1305 effects on the microtubule network were observed by immunofluorescence using a Confocal Scanning Laser Microscope (LSM) and Transmitted Light (TL). HTC 116 cells were incubated in the absence and the presence of C-1305 (10 or 20 μM) and paclitaxel (0.5 or 3 μM) for 24 h. Cells were fixed and processed for immunostaining with antibodies against $\alpha$-tubulin (left panel) and β-tubulin (right panel) for 2 h, and then reacted with Alexa fluoro-conjugated secondary antibody. The scale bar is 10 μm. Paclitaxel was used as the reference compound.

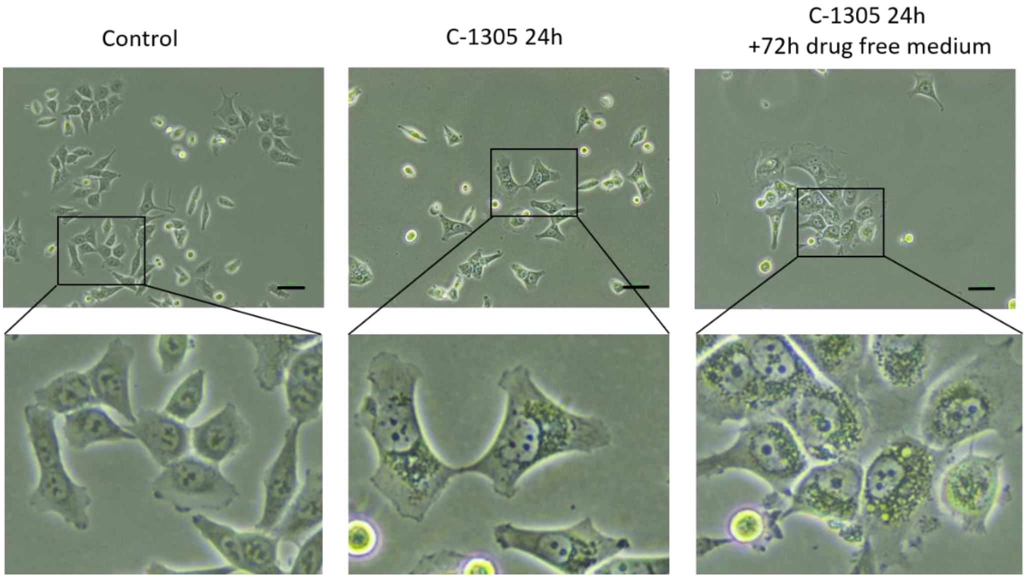


**Figure S11.** Cell morphology after C-1305 treatment. HCT 116 cells were treated with 10 μM C-1305 ($IC_{50}$ 10 μM) for the indicated time. The upper panel scale bars are 100 μm, lower panel shows the magnification of the indicated square from the upper panel. .

A.

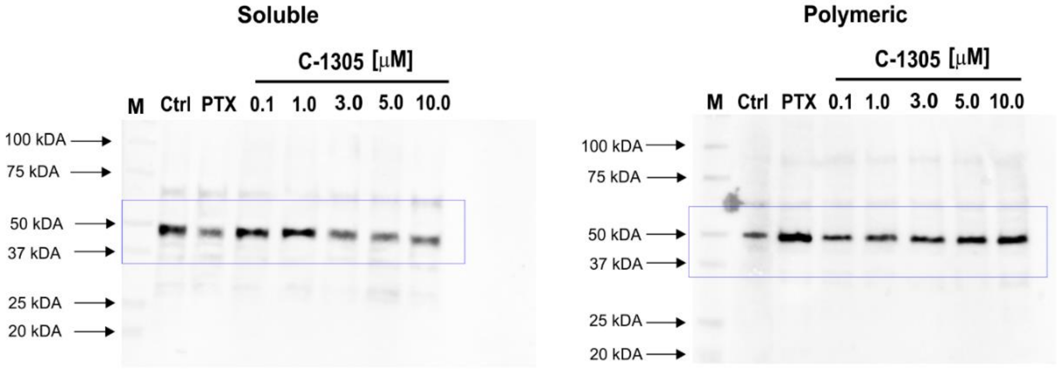


B. Densitometric analysis of Western blot

| Region | Total Density (OD) | Sample | |
|---|---|---|---|
| **Polymeric fraction** | | | |
| 1 | 627.61 | Ctrl | |
| 2 | 2500.80 | PTX | |
| 3 | 750.05 | 0.1 | C-1305 |
| 4 | 769.05 | 1.0 | C-1305 |
| 5 | 1065.51 | 3.0 | C-1305 |
| 6 | 1640.81 | 5.0 | C-1305 |
| 7 | 1909,20 | 10.0 | C-1305 |
| **Soluble fraction** | | | |
| 1 | 810.48 | Ctrl | |
| 2 | 370.17 | PTX | |
| 3 | 743.83 | 0.1 | C-1305 |
| 4 | 639.12 | 1.0 | C-1305 |
| 5 | 488.61 | 3.0 | C-1305 |
| 6 | 431.61 | 5.0 | C-1305 |
| 7 | 403.64 | 10.0 | C-1305 |

**Figure S12.** (**A**) Raw, not cropped supporting Western blot files of soluble and polymeric tubulin fraction in A549 cells after C-1305 treatment. A blue box denotes the cropped area shown in Figure 7A (main manuscript). To shown molecular weight marker (Precision Plus Protein™Kaleidoscope™ Prestained Protein Standards, Bio-Rad). Western blot was scanned with white EPI illumination before Chemiluminescent analysis and next merged. (**B**). Raw data used for relative mean band intensity calculation of different tubulin fraction after C-1305 treatment with respect to untreated cells presented in Figure 7A, (main manuscript).

© 2020 by the authors. Licensee MDPI, Basel, Switzerland. This article is an open access article distributed under the terms and conditions of the Creative Commons Attribution (CC BY) license (http://creativecommons.org/licenses/by/4.0/).
